# Kuwanon G Preserves LPS-Induced Disruption of Gut Epithelial Barrier In Vitro

**DOI:** 10.3390/molecules21111597

**Published:** 2016-11-22

**Authors:** Hengli Guo, Youhua Xu, Wei Huang, Hua Zhou, Zhaoguang Zheng, Yonghua Zhao, Bao He, Tingting Zhu, Shanshan Tang, Quan Zhu

**Affiliations:** 1 Faculty of Chinese Medicine, Macau University of Science and Technology, Avenida Wai Long, Taipa, Macao, China; henry162037601@gmail.com (H.G.); huangwei1212520@163.com (W.H.); hzhou@must.edu.mo (H.Z.); yhzhao@must.edu.mo (Y.Z.); zhulinke2012@163.com (T.Z.); luckyshaniaya@vip.sina.com (S.T.); 13570056996@163.com (Q.Z.); 2 State Key Laboratory of Quality Research in Chinese Medicine, Macau University of Science and Technology, Avenida Wai Long, Taipa, Macao, China; 3 Affiliated Hospital of Southwest Medical University, Luzhou 640000, China; 4 Macau Institute for Applied Research in Medicine and Health, Avenida Wai Long, Taipa, Macao, China; 5 Institute of Consun Co. for Chinese Medicine in Kidney Diseases, Guangdong Consun Pharmaceutical Group, Dongpeng Road 71, Guangzhou 510760, China; dzhg168@126.com (Z.Z.), hb_consun@163.com (B.H.)

**Keywords:** diabetes mellitus, gut epithelial barrier, inflammation, Kuwanon G, *Morus alba* L.

## Abstract

Defects in the gut epithelial barrier have now been recognized to be responsible for diabetic endotoxemia. In everyday life, Mulberry leaf tea is widely used in Asian nations due to its proposed benefits to health and control of diabetes. Evidence indicates the potential role of Kuwanon G (KWG), a component from *Morus alba* L., on blocking the gut epithelial barrier. In lipopolysaccharides (LPS)-damaged Caco-2 cells, it was found that KWG increased the viability of cells in a concentration-dependent manner. KWG administration significantly elevated the anti-oxidant abilities via increasing ratio of superoxidase dismutase (SOD)/malondialdehyde (MDA) and decreasing reactive oxygen species (ROS) within the cells. During KWG incubation, pro-inflammatory cytokines including interleukin (IL)-1β and tumor necrosis factor (TNF)-α were significantly reduced, tight junction proteins including zonula occludens (ZO)-1, intercellular adhesion molecule (ICAM)-1 and Occludin were dramatically increased as detected by immunofluorescence assay, trans-epithelial electrical resistance was significantly increased and the transmission of albumin-fluorescein isothiocyanate (FITC) across the barrier was decreased. In conclusion, the present study demonstrated that KWG could ameliorate LPS-induced disruption of the gut epithelial barrier by increasing cell viability and tight junction between cells, and decreasing pro-inflammatory cytokines and oxidative damage.

## 1. Introduction

Diabetes mellitus (DM) has become a worldwide problem that seriously weakens the quality of life in this population. Chronic low-grade inflammation has been recognized as one of the characteristics of DM, and this inflammation has been demonstrated to significantly exacerbate insulin resistance and the loss of islet β-cells [1]. In 1993, Hotamisligil and colleagues [2] firstly found in diabetic animals that neutralizing tumor necrosis factor (TNF)-α is helpful in ameliorating insulin resistance, indicating the important role of inflammation in the initiation and development of DM. Although the underlying mechanism is not fully elucidated, accumulating evidence highlights the gastrointestinal tract as a major source of chronic systemic inflammation at present.

The gastrointestinal tract constitutes an important part of the innate human immune system and is recognized to play a pivotal role in immediate protective response against pathogen invasion within the gut lumen [3,4]. The gut innate immune system is mainly composed of the epithelial cell barrier and the immune cells (e.g., neutrophils and natural killer cells). It is proposed that the defects of the cell barrier will lead to a paracellular influx of luminal antigens and toxins [5] and result in gut-sourced systemic inflammation. Although the adaptive immune system may counteract the pathogen and entoxin’s aggression to some extent under these settings, the overall effect is largely discounted. On the other hand, with the recognition of microbiota, it is suggested that gut dysbacteriosis should be the source of diabetic-endotoxemia [1]. Studies in both animal models and the human population have confirmed that gut dysbacteriosis will happen [6,7] and endotoxin-producing bacteria will increase [6] under DM settings. Studies found that endotoxin, with lipopolysaccharides (LPS) as representatives that originate from the cell wall of Gram-negative bacteria, is correlated with the severity of systemic inflammation [8,9]. As is well-known, LPS is responsible for multiple negative activities in the organism, including breaking down the integrity of different kinds of biological barriers. Therefore, strengthening the integrity of the gut epithelial barrier and inhibiting lumen LPS production or functions will help to ameliorate diabetic-endotoxemia.

*Morus alba* L. (Mulberry leaf) has been applied as a traditional herbal medicine to treat DM for hundreds of years both in China and worldwide [10,11,12]. In everyday life, Mulberry leaf tea is widely used in Asian nations due to its proposed benefits to health and control of diabetes [13]. Studies found in Mulberry leaf extract (MLE) can confront oxidative damage, decrease blood glucose and improve insulin resistance [12,14]. A recent study suggests that MLE has anti-microbial activity [15]. Moreover, MLE administration is helpful for decreasing the secretion of pro-inflammatory cytokines (e.g., TNF-α and monocyte chemoattractant protein (MCP)-1) [16] and inhibiting activation of nuclear factor-kappaB (NF-kappaB) [17] in diabetic animals. However, the specific component and mechanism responsible for its anti-diabetic inflammation is not known.

**Figure 1:**
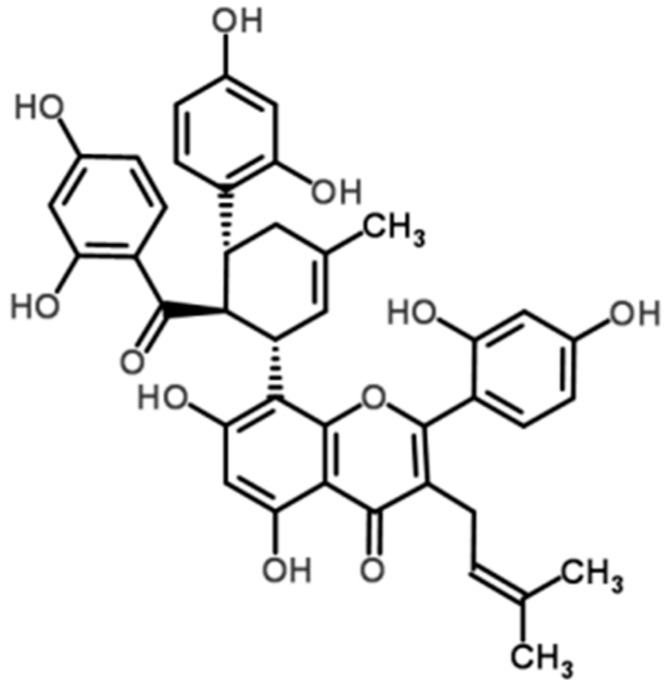
Chemical structure of Kuwanon G (KWG).

Flavonoids are an important class of components that exist in *Morus alba* L. and have been studied in detail due to their multiple biological activities. It is found that many flavonoids have properties against inflammation both in vivo and in vitro, and they may reduce the severity of many inflammatory diseases [18,19]. Since Galsanov and colleagues [20] reported the protecting effect of quercitrin (a flavonoid) against intestinal inflammation for the first time, more and more flavonoids are recognized to possess this activity [21]. Kuwanon G (KWG, Figure 1), a flavonoid with low bioavailability, is an important component of *Morus alba* L. Available evidence suggest that KWG may have beneficial effects on preserving the integrity of the gut epithelial barrier against inflammatory damage under DM conditions. Firstly, it was found that KWG possessed an activity against oral pathogens [22]; as pathogens always share some common characters, it is reasonable to postulate that KWG may rebalance the hemostasis of microbiota under DM settings. Another research work reported that KWG has antioxidant ability [23], and thus may ameliorate the oxidative stress state under DM settings. Previously, Jung and colleagues [24] found that KWG could inhibit the infiltration of inflammatory cells in ovalbumin (OVA)-induced allergic mice models of asthma and decrease pro-inflammatory cytokine secretion. The most recent research work indicated that KWG possessed a median effective concentration (EC_50_) of 0.8 ± 0.04 mg/L and median lethal concentration (LC_50_) of 38.0 ± 0.82 mg/L against theronst, and could be applied as a safe and effective drug to control ichthyophthiriasis [25]. From converging lines of evidence, we proposed a hypothesis that KWG may be a component that is responsible for the effect of Mulberry leaf against DM inflammatory damage. To demonstrate it, an in vitro gut epithelial barrier model was constructed in the present study, human epithelial colorectal adenocarcinoma (Caco-2) cells were applied, the influence of KWG on LPS-induced epithelial barrier disruption was detected, and the potential mechanism was investigated.

## 2. Results

### 2.1. Kuwanon G Dose-Dependently Increased the Viability of Caco-2 Cells

**Figure 2:**
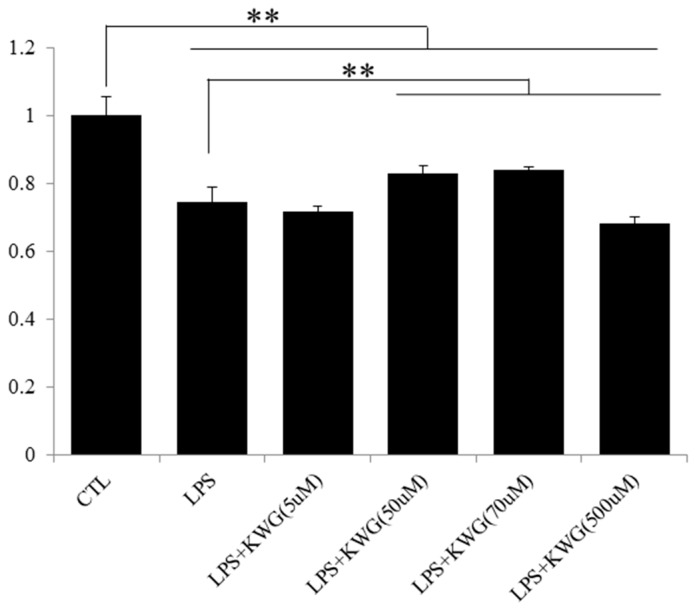
KWG dose-dependently reversed tumor necrosis factor (LPS)-damaged viability of Caco-2 cells. Cells were cultured in 96-well plates and were treated with 100 μg/mL LPS for 30 min followed by different concentrations of KWG for 24 h. The cell viability was detected by an 3-(4,5-Dimethylthiazol-2-yl)-2,5-diphenyltetrazolium bromide (MTT) method. The experiment was repeated six times. ** *p* < 0.01.

A recent finding from Song and colleagues [26] reported that LPS administration will induce epithelial cell apoptosis, the intestinal histological damage and gut leak in mice. Flavonoids have been widely reported to promote the proliferation of cells. In the current study, we found that KWG concentration dependently increased the viability of LPS-damaged Caco-2 cells (Figure 2). The most significant effect of KWG was observed at 70 μM. However, once the concentration of KWG was higher than 500 μM, an inhibition effect of KWG cell proliferation was shown. Therefore, we applied 70 μM to investigate effects of KWG on the cells in the present study.

### 2.2. KWG Inhibited LPS-Induced Secretion of Inflammatory Cytokines

**Figure 3:**
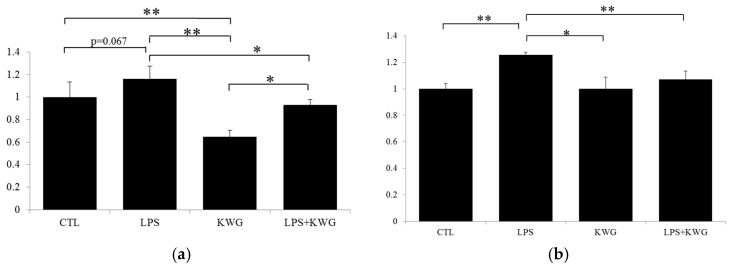
KWG decreased inflammatory cytokines’ expression in Caco-2 cells induced by LPS. Cells were treated with control (CTL), LPS, KWG or LPS + KWG for 24 h and the culture supernatant was collected. Inflammatory cytokines including (**a**) interleukin (IL)-1β and (**b**) tumor necrosis factor (TNF)-α were analyzed by ELISA kit according to the manufacturer’s instructions. * *p* < 0.05; ** *p* < 0.01.

Pro-inflammatory cytokines such as TNF-α and interleukin (IL)-1β will enhance the inflammatory response by promoting the recruitment and activation of other inflammatory elements, thus increasing the production and release of inflammatory mediators [27]. In the present study, we found 100 μg/mL LPS incubation dramatically increased the secretion of inflammatory cytokines compared with control (IL-1β, *p* = 0.067; TNF-α, *p* < 0.01) (Figure 3). In addition, KWG treatment could significantly reverse effects of LPS on the secretion of IL-1β and TNF-α to the normal levels.

### 2.3. KWG Increased Anti-Oxidative Stress Ability of Caco-2 Cells Induced by LPS

**Figure 4:**
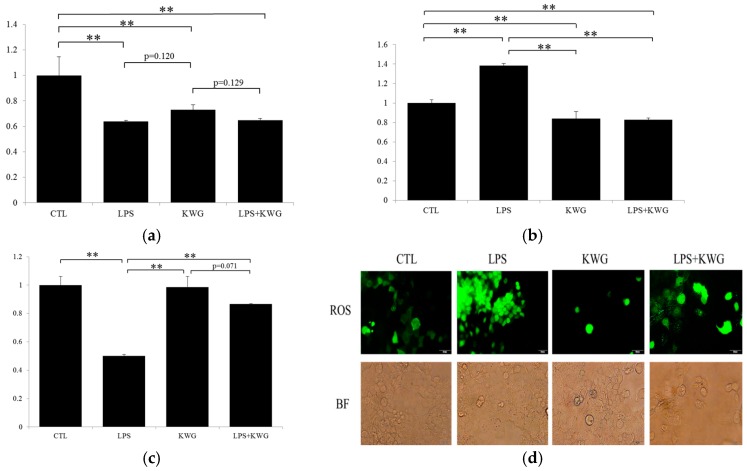
KWG strengthened anti-oxidative stress ability of Caco-2 cells against LPS-induced cell damage. Cells were treated with control (CTL), LPS, KWG or LPS + KWG for 24 h and the culture supernatant was collected. (**a**) Superoxidase dismutase (SOD) and (**b**) malondialdehyde (MDA) were detected by the kits according to the methods suggested by the manufacturer. (**c**) The ratio of SOD/MDA was analyzed to evaluate the overall anti-oxidative stress ability of the cells after different treatment. (**d**) Reactive oxygen species (ROS) were detected by the kit under fluorescence microscope, the corresponding cell morphology was captured under bright field (BF) (magnification: 200). The experiments were repeated for three times and representative figures or pictures were shown. ** *p* < 0.01.

Oxidative stress has been well known to play a negative role in cell activity. Once levels of pro-oxidant agents are over-generated or anti-oxidant agents are decreased, oxidative damage will occur. Oxidative damage plays an important role in the development of gut epithelial barrier leakage. It is reported that LPS could induce an oxidative stress state in different kinds of diseases [28]. In the current study, we found that, although treatment with KWG could not reverse LPS-induced decrease of superoxidase dismutase (SOD) level in Caco-2 cells (Figure 4a), it significantly decreased the levels of malondialdehyde (MDA) (Figure 4b). To comprehensively evaluate the anti-oxidative stress ability against LPS, the ratio of SOD/MDA was calculated. As depicted in Figure 4c, KWG administration significantly increased the ratio of SOD/MDA to the normal level.

Free radicals are a series of highly reactive molecules that can directly damage cell viability and function. There is a recent investigation suggesting that free radical molecules-induced cell damage may precede inflammatory process [29]. Reactive oxygen species (ROS) is an important component in the free radical family. Here, we applied a detection kit to observe the role of KWG in ROS. As shown in Figure 4d, LPS-induced ROS elevation was strikingly decreased after KWG treatment. Converging evidence from SOD, MDA and ROS analysis supports our hypothesis that KWG has satisfactory effects against LPS-induced oxidative damage in epithelial cells.

### 2.4. KWG Upregulated Intercellular Junction Protein Expression

**Figure 5:**
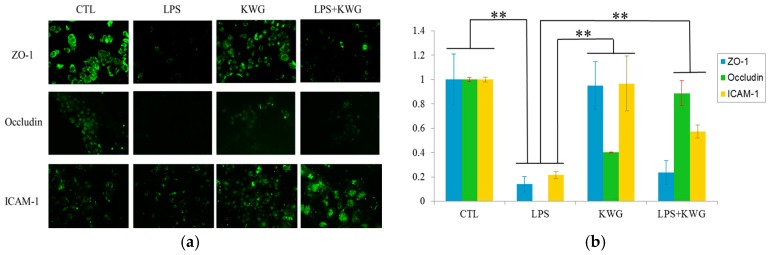
KWG upregulated the intercellular tight junction proteins’ expression. (**a**) Caco-2 cells were treated with control (CTL), LPS, KWG or LPS + KWG for 24 h, intercellular junction proteins including zonula occludens (ZO)-1, Occludin and intercellular adhesion molecule (ICAM)-1 were detected by the immunofluorescence method (magnification: 200). (**b**) Relative protein expressions were analyzed by Image-pro Plus software. The experiments were repeated three times and representative pictures were shown. ** *p* < 0.01.

The integrity of the gut epithelial barrier depends on the sufficient quantity of cells on the one hand, and functional tight junction between epithelial cells on the other hand [30]. To investigate if KWG could promote tight junction protein expression, intercellular junction proteins including zonula occludens (ZO)-1, Occludin and intercellular adhesion molecule (ICAM)-1 were detected by the immunofluorescence method, and the relative protein expression level was analyzed by Image-Pro Plus software (Media Cybernetics, Rockville, MD, USA). As shown in Figure 5, LPS decreased expression of intercellular junction protein by as high as 80%, while when the cells were administrated with KWG, expressions of Occludin and ICAM-1 were significantly increased compared with the LPS group; however, we did not find that KWG treatment can reverse LPS-induced ZO-1 decrease.

### 2.5. KWG Strengthened Gut Epithelial Barrier Integrity in LPS Circumstances

**Figure 6:**
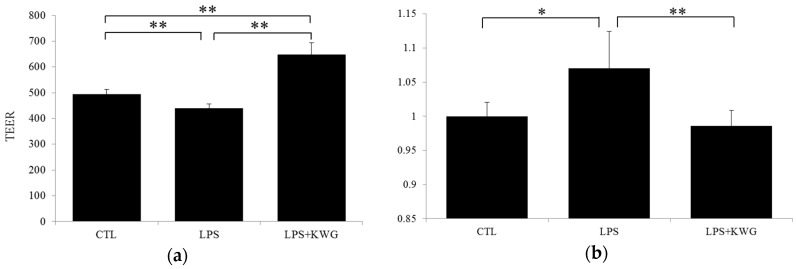
KWG reversed LPS-induced gut epithelial cell barrier defect in Caco-2 cells. (**a**) the trans-epithelial electrical resistance (TEER) was measured after the cells were treated with control (CTL), lipopolysaccharide (LPS) or LPS + KWG for 24 h. (**b**) albumin-fluorescein isothiocyanate (FITC) across the epithelial barrier was detected by a microplate reader at the wavelength of 495 nm. The experiment was repeated three times at different time points. * *p* < 0.05, ** *p* < 0.01.

To comprehensively evaluate the influence of drug administration on gut epithelial barrier integrity, we analyzed trans-epithelial electrical resistance (TEER), and trans-epithelial albumin- fluorescein isothiocyanate (FITC). As shown in Figure 6a, LPS decreased TEER by 10.86% (*p* < 0.01, vs. control), while KWG administration significantly increased TEER to the level even higher than that of normal (*p* < 0.01, vs. control and LPS). These data strongly support our hypothesis that KWG could enhance the physical barrier of the gut epithelial cells. To further demonstrate our findings, we evaluated large-molecular substance permeability across the epithelial barrier. Albumin is a large molecular with molecular weight of 66 kd. For the detection purposes, we obtained albumin that is conjugated with FITC. We found that KWG administration strikingly decreased the content of albumin in the bottom well to that of the normal level (*p* < 0.01, vs. LPS) (Figure 6b).

## 3. Discussion

Diabetic endotoxemia has been now recognized as a characteristic which seriously brings DM into a tight spot. In this decade, it has been recognized that gut-sourced LPS plays a pivotal role in this process [1]. Accumulating evidence brings us the possibility that inflammation-target treatment may help to prevent the development of diabetic endotoxemia. In the present study, we found for the first time that Kuwanon G, a flavonoid, can preserve gut epithelial barrier integrity by increasing the viability of epithelial cells and protecting cells from LPS-induced oxidative and inflammatory damage.

It has been well recognized that intestinal epithelial cells (IECs) function play a pivotal role as a natural barrier against luminal contents invading the underlying tissues [31]. The integrity of this barrier recurs enough of the cells and tight junctions between them, and any inference towards these two factors will induce the disturbance of the gut epithelial barrier and induce the so-called “gut leak”. In this case, pathogens or toxins within the gut lumen may easily invade across this incomplete barrier into the underlying tissue and result in systemic inflammation [3,4]. In this sense, either limiting the damage to epithelial cells or strengthening the activity of the cells will have protective effects for this barrier.

Since Hotamisligil and colleagues [2] explored the significance of neutralizing pro-inflammatory cytokines on ameliorating DM, the term “diabetic endotoxemia” is suggested. However, the source that contributes to this is not well illustrated until the discovery of the important role of microbiota in the development of DM can be found [7]. It is found that dysbacteriosis under DM settings will dramatically increase the production of LPS within the gut lumen [6] and thereafter induce a series of damages to the gut epithelial barrier. There is a report that suggests that a unique defect in this barrier is enough to trigger the development of chronic gut inflammation [32]. As the impairment of the epithelial barrier will facilitate the entrance of pathogens or toxins from the intestinal lumen to the mucosa, which will result in the uncontrolled and exacerbated immune response, it is currently recognized as one of the early events that occurs in intestinal inflammation [33,34].

In the present study, we observed that LPS administration significantly decreased the viability of Caco-2 cells. We believe this reduction constitutes a basis that attributes to “gut leak”. This hypothesis is supported by a recent finding from Song and colleagues [29] that LPS administration will induce epithelial cell apoptosis, intestinal histological damage and gut leak in mice. Conventionally, it is believed that effects of LPS on Caco-2 cells may be “moderate” due to a lack of TLR4 in the cells. Most recent reports demonstrate that there is TLR4 expression in Caco-2 cells and thus Caco-2 cells can respond to LPS [35,36]. We observed in our preliminary test that the viability of Caco-2 cells was not significantly influenced when they were treated with relatively low concentration of LPS (10~50 μg/mL); however, once the concentration was higher than 100 μg/mL, a significant anti-proliferation effect of LPS was observed (data not shown). In this sense, a “moderate” effect can only be a phenomenon in that LPS at low concentration. This also explains the reason why the gut epithelial barrier can keep its integrity in healthy populations: although there are Gram-negative bacteria (e.g., *E. Coli*) and LPS exist within the gut lumen, they are at low level.

Consistent with previous reports, we also observed in our present study that LPS promoted the secretion of pro-inflammatory cytokines and weakened the anti-oxidant capacity of epithelial cells. It has been demonstrated that cytokines such as TNF-α and IL-1β will enhance the inflammatory response by promoting the recruitment and activation of other inflammatory elements, thus increasing the production and release of inflammatory mediators [27]. Moreover, the activation of inflammatory cells will exacerbate oxidative stress process, and oxidative stress can directly damage cell membrane or important genetic material and finally induce cell apoptosis. Therefore, apoptosis-inflammation-oxidative stress constitutes a vicious cycle, and disturbing any component of this cycle will help to inhibit LPS-induced gut leak.

*Morus alba* L. (Mulberry leaf) has been applied as a traditional herbal medicine to ameliorate DM and its related complications for hundreds of years both in China and worldwide [10,11,12]. Previously, Mulberry leaf extract (MLE) was shown to have anti-hyperglycemic, anti-oxidant, and anti-glycolic properties, and improve insulin resistance activities in diabetic animals [14,37,38]. Moreover, it was found to have anti-inflammation properties. Studies demonstrated that MLE treatment could decrease the expression of TNF-α, MCP-1 and macrophage markers [16], and inhibit TNF-α-induced activation of nuclear factor-kappaB (NF-kappaB) [17]. Another piece of research found that MLE had antimicrobial activities [15]. Available evidence shows us that the modulation on gut-sourced inflammation may be an important mechanism that contributes to therapeutic effects of *Morus alba* L. on DM. However, the specific components and mechanisms are yet to be elucidated.

Flavonoids are a class of compounds that are found to be enriched in *Morus alba* L., and many flavonoids have been described to suppress inflammation [18,19,21]. Mechanisms concerning the intestinal anti-inflammatory activities of flavonoids mainly include antioxidant properties, immunomodulatory activity, and preservation of the gut epithelial barrier functions [39,40]. Increasing evidence supports the role of microbiota in the initiation and development of the intestinal inflammatory process [41,42,43,44], while diets containing flavonoids can be considered as possible complementary treatment for intestinal inflammatory disease due to their anti-microbial and anti-oxidant capacities [45,46]. There is also a study [47] suggesting that phenolic compounds possess selectively bacteriostatic or bactericide effects, and they can inhibit the growth of a wide range of potential pathogenic bacteria while slightly affecting or even promoting the beneficial microbial population. There is a report that demonstrated that incubation of LPS-activated inflammatory cells with quercetin would result in a significant reduction of IL-1β and TNF-α compared with non-flavonoid treatment [48]. However, as the number of reported flavonoids on this effect is relatively small, the structure-activity relationship is not well illustrated and it is still to be clarified if this effect is attributed to their local or systemic function [40]. To demonstrate our hypothesis and investigate the potential mechanism, we systemically analyzed components of *Morus alba* L. and obtained a flavonoid, KWG, by cyto-membrane immobilized chromatography.

To investigate the role of KWG on LPS-induced disruption of the gut epithelial barrier in vitro, we found that KWG administration can significantly ameliorate LPS-induced epithelial cell damage by increasing cell viability, enhancing cell anti-oxidant ability and inhibiting secretion of pro-inflammatory cytokines. Our present findings are obviously in accordance with previous reports. A research work reported that KWG has antioxidant ability [23]. Previously, Jung and colleagues [24] found that KWG could inhibit the infiltration of inflammatory cells in ovalbumin (OVA)-induced allergic mouse model of asthma, and decrease OVA-specific IgE and IL-4, IL-5, and IL-13 in the sera and bronchoalveolar lavage fluids. The most recent research work indicated that KWG possessed a median effective concentration (EC_50_) of 0.8 ± 0.04 mg/L and a median lethal concentration (LC_50_) of 38.0 ± 0.82 mg/L against theronst, and could be applied as a safe and effective drug to control ichthyophthiriasis [25]. It should be noted that we didn’t observe a significant effect of KWG on elevating SOD activity in our present study; moreover, KWG reduced SOD activity compared with control; furthermore, KWG application after LPS incubation decreased SOD level further, although no significance was observed (*p* = 0.129, KWG vs. LPS + KWG). We should say that this is indeed an unexpected phenomenon from our hypothesis, and the exact mechanism should be further explored, as available data and references are few and cannot explain this phenomenon. However, we propose that the intracellular signaling mechanism may be involved in KWG-modulated expression of SOD. Although results from SOD tests were not in accordance with our expectations, our findings that KWG can elevate the ability of cells to confront LPS-induced oxidative damage is not discounted, as KWG application decreased the level of MDA, increased the ratio of SOD/MDA, and reduced ROS production.

As discussed above, the major function of the intestinal barrier is to protect the organism against invading pathogens or toxins [3,4], while defects in the barrier will result in a paracellular influx of luminal antigens and toxins [5]. Besides cell quantity, tight junctions between cells are also essential for the barrier integrity. The apical junction complex of gut epithelial barrier is composed of transmembrane proteins including claudins, occludin, E-cadherin and cytoplasmic linker proteins [49]. It is reported that depletion of these proteins will result in the leaky barrier [50]. With the increasing understanding of the importance of the gastrointestinal tract ecosystem in systemic disease, it is recognized that the leaky barrier contributes a lot to the development of DM [51]. Previous studies in diabetic animals have demonstrated that hyperglycemia is correlated with the reduction of tight junction proteins [52,53]. Both in vivo and in vitro studies have observed that LPS administration can increase cell apoptosis, decrease tight junction proteins’ expression, induce intestinal histological damage, and markedly increase the paracellular permeability and gut leak [29,54]. It is also observed in our present study that LPS significantly decreased TEER across the epithelial barrier; moreover, the permeability of large molecules (albumin-FITC in this paper) is dramatically increased in LPS administration. KWG expectedly preserved TEER and inhibited LPS-induced epithelial barrier disruption by increasing tight junction protein expression [39]. It has been reported that inflammatory cytokines may seriously affect cell viability and thus disrupt the epithelial barrier function [55,56]. In this sense, the inhibitory effect of KWG on pro-inflammatory cytokine secretion also contributes to the improvement of intestinal permeability [57]. Interestingly, we observed in the present study that KWG application alone decreased expression of occludin. As available data and references are limited, we cannot explain this phenomenon well at present, and we may explore the exact underlying mechanism further.

In the present study, we observed that KWG alone had significant effects on decreasing inflammatory cytokines’ secretion, strengthening the overall anti-oxidative damage of the cells, and increasing tight junction protein expression between gut epithelial cells. On the other hand, abundant evidence has demonstrated the role of flavonoids in promoting cell proliferation. Converging with the other research groups’ reports and our present findings, it is reasonable to postulate that KWG alone would also increase TEER and thus increase gut barrier integrity; in this sense, daily application of Mulberry leaf tea may have a good effect on preserving gut health, and its component KWG should play a significant role in this effect. However, as the available paper involving Kuwanon G is short (only nine papers were found in Pubmed until now), more work is necessary to fully explore the effects of KWG on diabetic endotoxemia. There is a previous finding reported that KWG possessed activity against oral pathogens [22]. On the other hand, as KWG is a phenolic compound, most of it may reach and accumulate in the intestine lumen after oral administration due to its low bioavailability [58]. In this sense, systemically investigating the influence of KWG on microbiota under DM settings is necessary.

## 4. Experimental Section

### 4.1. Materials

Kuwanon G was supplied by Pufeide Biotech Co., Ltd. (Chengdu, China). Lipopolysaccharide (LPS), which is derived from *Escherichia coli* 055:B5, was purchased from Sigma (St. Louis, MO, USA; product number: L2880). Dulbecco’s modified eagle medium (DMEM), Fetal Bovine Serum (FBS), trypsin, MEM Non-Essential Amino Acids Solution, and L-Glutamine were obtained from Gibco (Big Cabin, OK, USA). 3-(4,5)-dimethylthiazo(-z-y1)-3,5-diphenyt-etrazoliumromide (MTT), ELISA kits for IL-1β and TNF-α were purchased from Neobioscience (Shenzhen, China). Primary antibodies against Zona Occludens protein-1 (ZO-1), Intercellular Adhesion Molecule-1 (ICAM-1) and Occludin were purchased from Santa Cruz (Dallas, TX, USA). FITC-conjugated secondary antibody was supplied by Boster (Wuhan, China). In addition, 24-well Transwell cell culture plates (hanging insert well diameter 6.5 mm, membrane area 0.3 cm^2^) were obtained from Corning (Corning, NY, USA). The electrical resistance detection system (Millicell ESR-2) was bought from Millipore (Billerica, MA, USA). The other materials were from commercial sources.

### 4.2. Cell Culture

Human epithelial colorectal adenocarcinoma (Caco-2) cells were obtained from the American Type Tissue Collection (Manassas, VA, USA). The cells were cultured according to the method described by Akbari and colleagues [59]. In general, the cells were cultured in DMEM medium supplemented with 10% heat-inactivated fetal bovine serum (FBS), penicillin (100 units/mL), streptomycin (100 μg/mL), 1% MEM Non-essential amino acids and L-Glutamine at 37 °C in a humidified atmosphere of 5% CO_2_/95% air.

### 4.3. Cell Viability Evaluation by MTT Assay

Caco-2 cells at exponential phase were seeded in flat-bottom 96-well cell culture plates. Cells were administrated with vehicle (as control), LPS, KWG, or LPS + KWG as indicated for 24 h. Then, cells were incubated with 3-(4,5)-dimethylthiazo(-z-y1)-3,5-diphenyt-etrazoliumromide (MTT) (5 mg/mL) for 4 h in the incubator. Formazan was dissolved by Dimethyl Sulphoxide (DMSO). A spectrophotometer (TECAN, Seestrasse, Switzerland) was used to test the absorbance at wavelength of 490 nm.

### 4.4. ELISA for Evaluation of Inflammatory Cytokine Expression

Caco-2 cells were incubated with vehicle (as control), LPS (100 μg/mL), KWG (70 μM), or LPS (100 μg/mL) + KWG (70 μM) for 24 h, and the cell culture supernatant was collected for evaluating levels of inflammatory cytokines including IL-1β and TNF-α by ELISA according to the manufacturer’s protocols.

### 4.5. Measurement of Cell Anti-Oxidant Activity

Caco-2 cells were seeded in 24-well cell culture plates and administrated with vehicle (as control), LPS (100 μg/mL), KWG (70 μM), or LPS (100 μg/mL) + KWG (70 μM) for 24 h, anti-oxidant activity of the cells were comprehensively evaluated by detecting oxidative stress factors including SOD, MDA and ROS by the detection kits according to the manufacturer’s protocol. In general, the culture supernatant was collected and SOD/MDA was analyzed by the spectrophotometer, and the level of ROS in the cells was observed by the immunofluorescence microscope.

### 4.6. Immunofluorescence Analysis

Caco-2 cells at exponential growth state were seeded on the slide in 12-well plates and were administrated with a vehicle (as control), LPS (100 μg/mL), KWG (70 μM), or LPS (100 μg/mL) + KWG (70 μM) for 24 h. After being gently washed with phosphate buffer saline (PBS) solution, the cells were fixed with 4% paraformaldehyde for 10 min. Then, the cells were washed with PBS three times and blocked with rabbit serum for 30 min at room temperature. Thereafter, the cells were incubated with primary antibodies including ZO-1 (1:100), Occludin (1:100) or ICAM-1 (1:100) for 1 h at room temperature followed by PBS washing three times (3 min/time). Secondary antibody conjugated with FITC was applied to observe the proteins’ expression under fluorescence microscope (Olympus, Tokyo, Japan). The relative expression of proteins was analyzed by Image-Pro Plus software (7.0, Media Cybernetics, Rockville, MD, USA).

### 4.7. Gut Epithelial Barrier Model Construction and Substance Permeability Evaluation

**Figure 7:**
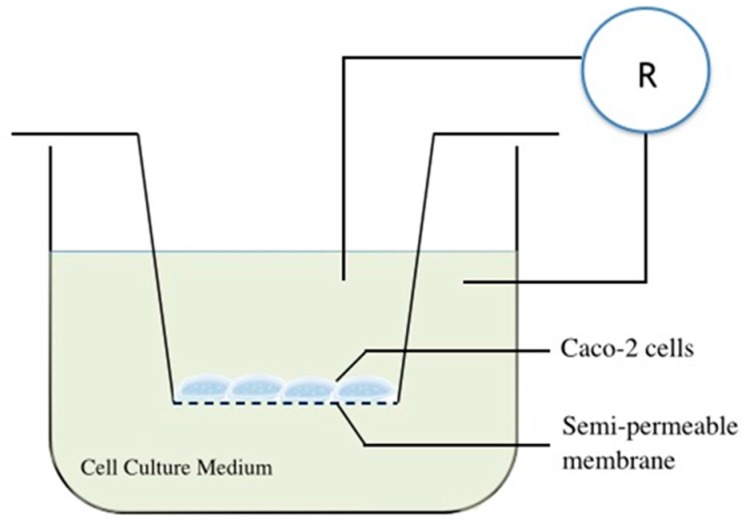
The schematic diagram of the gut epithelial barrier model construction in vitro.

An in vitro gut epithelial barrier model was constructed using cell culture inserts according to the method described previously [59]. In brief, Caco-2 cells were cultured in the medium and seeded at a density of 1 × 10^5^ cells into inserts with a polyethylene terephthalate membrane (pore diameter, 0.33 μm) plated in 24-well cell culture plates (Figure 7). Then, the cells were maintained in the cell incubator for 21 days for cell culturing, and a confluent monolayer was finally obtained. The TEER across the monolayers was measured with a Millicell-ERS electric resistance system (Millipore, Billerica, MA, USA) [60], and the mean TEER will exceed 400 Ω · cm^2^ after 21 days of incubation [61].

To analyze the influence of drug administration on the permeability of large molecular substance across the epithelial barrier, the ability of albumin conjugated with FITC across the barrier was detected. Specifically, after Caco-2 cells were administrated with a vehicle (as control), LPS (100 μg/mL), or LPS (100 μg/mL) + KWG (70 μM) for 24 h, the incubation mixture in the upper and bottom well of the system was replaced with serum-free medium. Then the albumin-FITC (final concentration, 1 mg/mL) was added into the upper well of the system and the cells were further incubated for 2 h; finally, the concentration of albumin-FITC in the bottom well was detected by the microplate reader at a wavelength of 495 nm.

### 4.8. Statistical Analysis

The data were expressed as mean ± standard deviation (SD) for at least 3 independent experiments. Differences among groups were analyzed by SPSS 19.0 software (IBM, Chicago, IL, USA) by one-way ANOVA methods, and a post hoc Fisher's Least Significant Difference (LSD) test was used. *p* < 0.05 was regarded as statistical significance.

## 5. Conclusions

**Figure 8:**
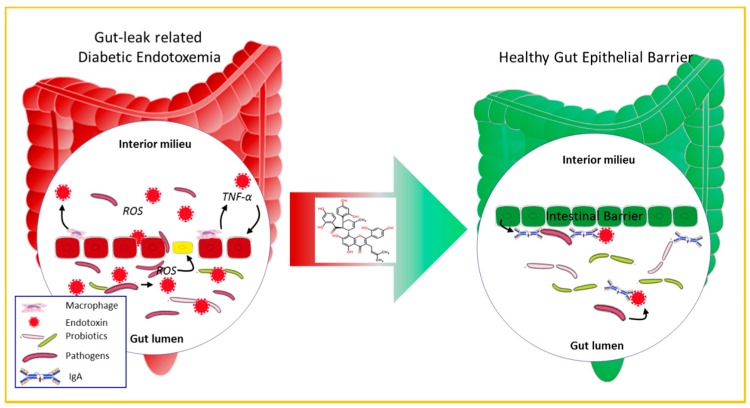
Mechanism of KWG on protecting gut epithelial barrier integrity.

In conclusion, we found for the first time that Kuwanon G possesses a beneficial effect of preserving the gut epithelial barrier against LPS-induced disruption (Figure 8). The mechanism lies in its effects on promoting cell proliferation, increasing cell anti-oxidant ability, and inhibiting pro-inflammatory cytokines. The present findings reveal in part the mechanism of *Morus alba* L. in treating DM and propose a novel strategy against diabetic endotoxemia and other intestinal inflammatory disease.
